# Topical dorzolamide treatment of macular cysts in the enhanced S-cone syndrome patient

**DOI:** 10.1007/s10633-012-9371-9

**Published:** 2013-01-06

**Authors:** Marta Kiszkielis, Wojciech Lubiński, Krzysztof Penkala

**Affiliations:** 1 Chair and Clinic of Ophthalmology, Pomeranian Medical University, Powstańców Wlkp. Street 72, 70-111 Szczecin, Poland; 2 Department of Systems, Signals and Electronics Engineering, West Pomeranian University of Technology, Szczecin, Poland

**Keywords:** Macular cysts, Enhanced S-cone syndrome, Foveal function and thickness, Topical dorzolamide

## Abstract

**Purpose:**

The purpose of the study was to evaluate the efficacy of a topical form of a carbonic anhydrase inhibitor (dorzolamide) on the foveal function and thickness in the eye of a patient with enhanced S-cone syndrome (ESCS) associated with macular cysts.

**Methods:**

Twenty-eight-year-old Polish man with ESCS and macular cysts appearance in the right eye was treated 3 times daily with 2.0 % dorzolamide drops for the period time equal to 6 months. Monthly controls included: best corrected distance visual acuity (BCDVA-logMAR), foveal thickness (optical coherence tomography, OCT) and foveal function (multi-focal electroretinography, mfERG).

**Results:**

Before treatment, BCDVA in the right eye was equal to 0.26 logMAR, improved to 0.1 logMAR during the first 3 months and remained stable for the next 3 months. After 6 months, foveal thickness decreased (from 482 to 224 *μ*m) and foveal function improved (the amplitude of P1-wave density increased from 34.8 to 107.3 nV/deg^2^) and was between the ranges of normal values. Implicit time of P1-wave remained prolonged.

**Conclusions:**

The results of our short-term study suggest potential efficacy of topical dorzolamide treatment in ESCS patients with macular cysts.

## Introduction

Enhanced S-cone syndrome (ESCS) first described in 1990 by Marmor et al. [1] is a rare, slowly progressive autosomal recessive inherited retinal degenerative disorder. In most cases of ESCS, NR2E3 gene mutation is responsible for the disease development and leads to abnormal cones and rods differentiation [2]. Characteristic fundus changes usually progress from subtle pigmentary changes with white dots in the early stage to a more severe nummular pigmentary deposition around the vascular arcades accompanied by yellowish dots later on. Cystic macular changes occur frequently, in about 50–75 % of cases [3, 4].

Enhanced S-cone syndrome is diagnosed on the basis of characteristic clinical fundus changes and pathognomonic features of the full-field electroretinography (ERG). There were performed according to the International Society for Clinical Electrophysiology of Vision (ISCEV) standards such as the absence of rod response, morphologically similar waveforms of scotopic and photopic responses to the standard stimulus flashes (3.0 ERG) and flicker reduction [4, 5]. The S-cone ERG recording can be performed to confirm the pathophysiologic origin of the disease (the S-cone hyperactivity) and is characterized by abnormally large and delayed waveforms (relative to those with standard stimulation) [3].

Retinal pigment epithelium (RPE) function is abnormal in ESCS retina [6], resulting in macular cysts development. Dorzolamide (topical form of carbonic anhydrase inhibitor) may improve RPE metabolism by activating the pumping function and consequently, it reduces intraretinal cysts and RPE remains adherent to the retina [7, 8].

Only 3 case report studies [9–11] described efficacy of topical (2.0 % dorzolamide) and/or oral (acetazolamide) carbonic anhydrase inhibitor in the treatment of macular cysts associated with ESCS. In these 3 patients, increase in visual acuity associated with normalization of foveal microanatomy (illustrated by OCT) was observed. Treatment time in these studies varied from 4 [9, 10] up to 27 months [11]. During the follow-up, visual acuity, foveal thickness and structure in each patient improved and remained stable. These promising initial results were the reason to implement dorzolamide therapy in a newly diagnosed ESCS patient presented in this report (Table 1).

## Case

A twenty-eight-year-old white male was sent to the ophthalmology clinic with the suspicion of ESCS and had following complaints: night vision problems since the childhood and decreased visual acuity since 2 years in the left eye and most recently in the right eye. Informed consent was obtained from the patient before the examination.

**Fig. 1:**
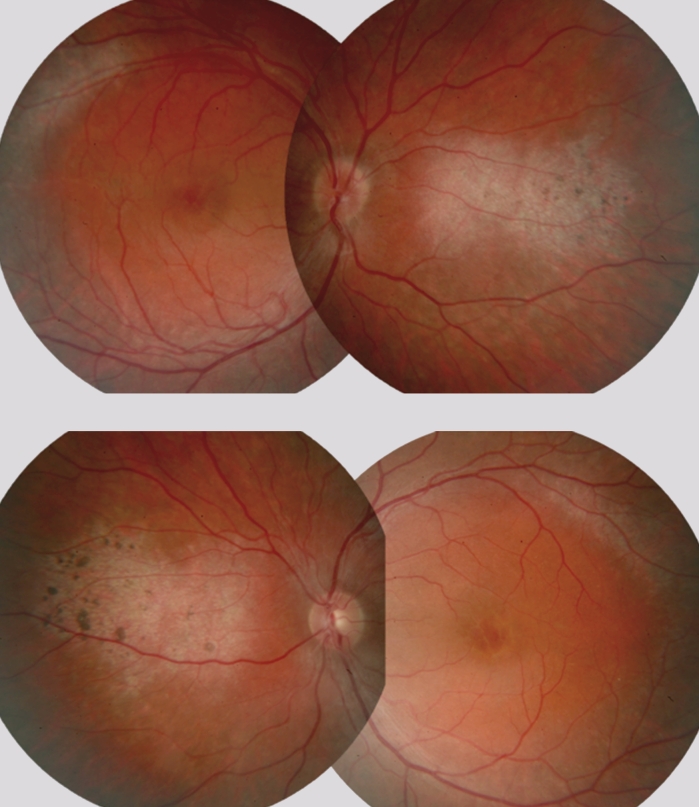
Fundus photographs, the right and left eye of the 28-year-old man with ESCS

**Fig. 2:**
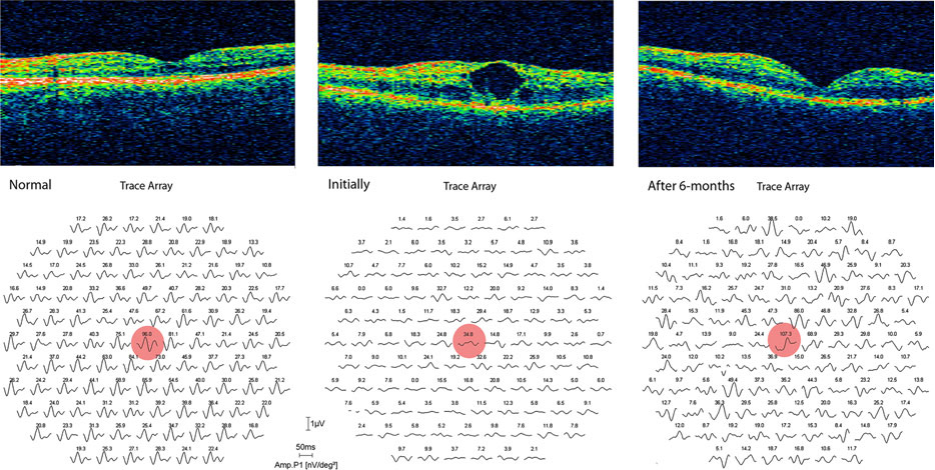
Optical coherence tomography (OCT) images (above) and mfERG recordings (below) of the right eye of the 28-year-old man with ESCS before and after a 6-month treatment with topical dorzolamide in comparison with the age-matched healthy control

Initially, the patient’s BCDVA was equal to 0.26 in the right eye and 0.2 in the left eye on a logMAR scale (early treatment diabetic retinopathy study chart). Color vision (Farnsworth Panel-15 D) was normal in both eyes. Fundus exam revealed white dots around the vascular arcades accompanied by single black flecks. Optic disc and vessels were normal. Clinical features of macular cysts were noticed in both eyes (Fig. 1). Macular cysts appearance was confirmed by the OCT test (Zeiss Stratus OCT, Humphrey Instruments model 3000, Carl Zeiss Inc, Dublin, CA, 2007). The cystoid spaces appeared as hyporeflective spaces, interspersed with high-signal septae which represented bridging retinal elements in both eyes. Loss of the normal foveal depression was observed in the right eye (Fig. 2). In the left eye, foveal contour became even convex and dome shaped, as previously described in the literature [6]. Fluorescein angiogram showed absence of any detectable leakage of the dye in the macular area in either eye.

**Fig. 3:**
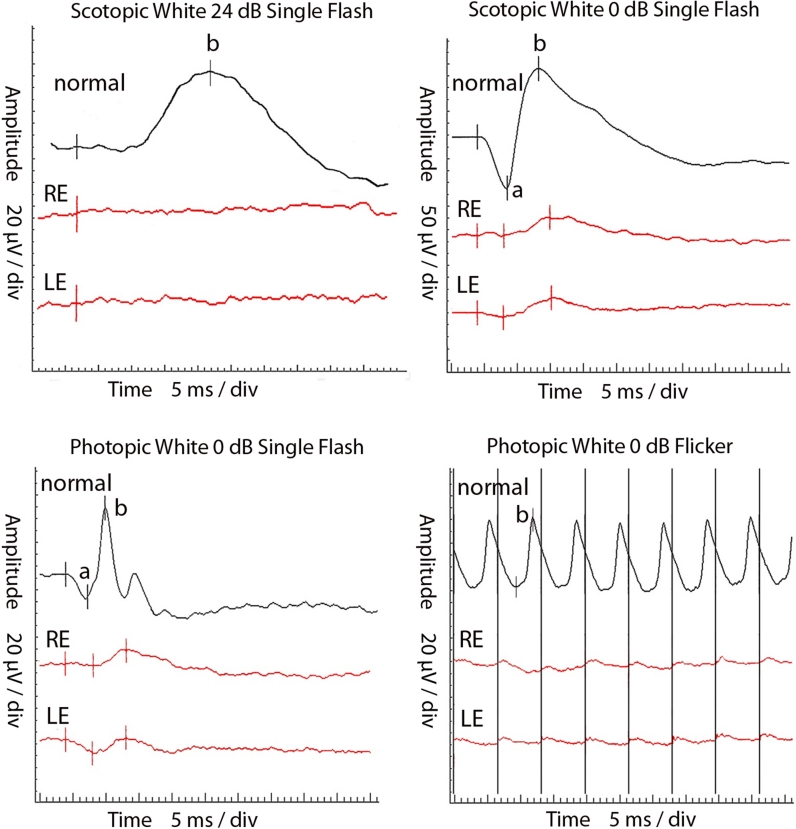
Full-field ERG of the ESCS patient

The ERG (ISCEV) [12] was recorded, using Burian–Allen bipolar electrodes (UTAS-E2000, LKC, USA). It showed the absence of rod response, reduced and delayed waveforms in response to white flash (0 dB), both in scotopic and photopic conditions, reduced and delayed flicker recording (Fig. 3). In the S-cone ERG recordings, blue stimulus (455 nm, 5 ms, 80 cd/m^2^, 16 cd s/m^2^) was used in the orange background (590 nm, 560 cd/m^2^). Parameters were similar to those performed by Audo et al. [3]. The recordings presented hyper-normal and delayed a- as well as b waves. In mfERG (ISCEV) [13] (RetiScan system, Roland Consult, Germany, 2009), delayed and reduced P1-wave amplitudes were observed in all six analyzed concentric rings. NR2E3 gene sequencing was not performed, because it is not necessary for ESCS diagnosis.

The patient was treated with topical form of 2.0 % dorzolamide 3 times daily for 6 months. Initially, it was applied to both eyes. Left eye treatment was discontinued after 1 month because of inefficacy and cystoid macular changes progression. The probable reason of the left eye treatment failure was prolonged macular cysts appearance, which caused profound and irreversible RPE and cone photoreceptors dysfunction.

Examinations that were performed after 1st, 2nd, 3rd, 4th and 6th months of the treatment consisted of: BCDVA, mfERG and OCT tests (5th month examination failed due to the absence of the patient). The tenets of the Declaration of Helsinki were followed. Informed consent was obtained after the details of the procedures had been fully explained.

**Fig. 4:**
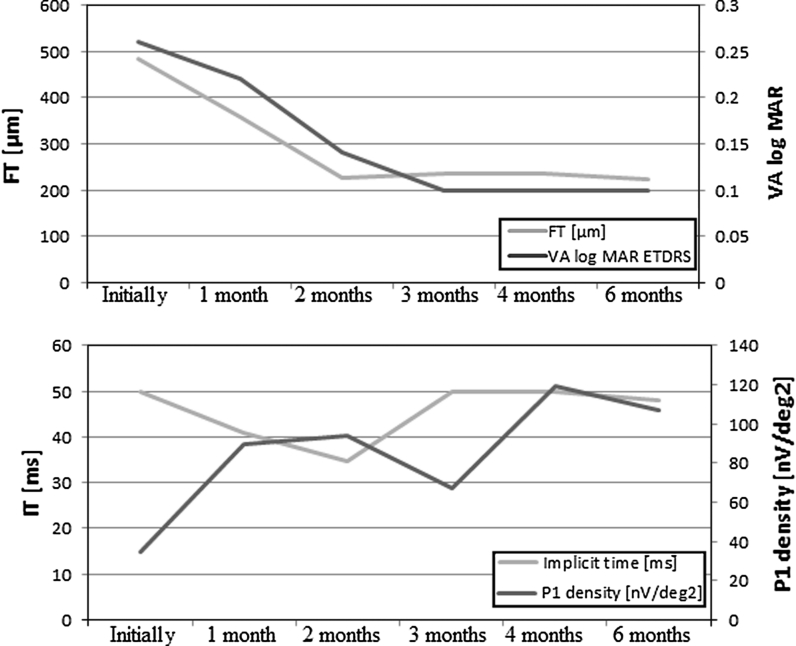
*Right eye* foveal thickness (FT) and visual acuity (VA) (*above*); implicit time (ms) and P1-wave response density (nV/deg^2^) of mfERG results (*below*) of ESCS patient, treated with dorzolamide during 6 consecutive visits

BCDVA increased gradually during the treatment from 0.26 to 0.22 logMAR at the 1st, to 0.14 logMAR at the 2nd and to 0.1 logMAR at the 3rd follow-up examination (Fig. 4). BCDVA in the right eye remained stable during the next 3 months of treatment. Except the 3rd follow-up examination, central retinal function, presented by P1 wave response density in mfERG improved and recover to normal value during 6 months follow-up. One of the possible explanations of this was unsystematic use of the drops in the 3rd month, which was confirmed by the patient. P1 wave implicit time during first 2 months of treatment shortened, but then it returned to the value before the treatment (Fig. 4).

**Table 1:** .

	VA logMAR ETDRS	FT (μm)		P1 density (nV/deg^2^)		Implicit time (ms)	
		M ± SD	Range	Med	Range	M ± SD	Range
Normal values	0.0	212 ± 20	192–232	134.5	106.8–148.2	39.2 ± 2.1	35–43.3

*M* mean, *SD* standard deviation, *Med* median

The improvement of BCDVA in the right eye corresponded with foveal thickness reduction from 482 *μ*m initially to 359 *μ*m at the 1st and to 226 *μ*m at the 2nd follow-up visit (Fig. 2). At this point, intraretinal cysts were completely absorbed. Foveal thickness remained unchanged for the next 3 follow-up visits.

## Discussion

The main reason behind the decreased visual acuity in ESCS patients is cystoid changes in macular region. Macular cysts early diagnosis may be of great value for ESCS patients, when at least partial RPE function is preserved. It is possible that by using topical dorzolamide, which provides an increased RPE pumping function and in consequence intraretinal fluid absorption, restoration of normal foveal morphology can be obtained and even in some cases, visual acuity may improve [9–11].

An extracellular membrane-bound carbonic anhydrase (CA XIV) was shown to be present in brain and retina (astrocytes, Muller cells and RPE). Carbonic anhydrase XIV is the target of carbonic anhydrase inhibitors that enhance subretinal fluid absorption in macular edema [14]. Carbonic anhydrase inhibitors usefulness was proven in the treatment of macular cystoid changes associated with diseases such as retinitis pigmentosa [15–18], choroideremia [19] and X-linked retinoschisis [20].

To our best knowledge, this is the third case report study describing topical dorzolamide efficacy in macular cysts management in the ESCS patient, with the longest follow-up period equal to 6 months. Dorzolamide treatment was applied, because we assumed that cystoid macular spaces were filled with endogenous interstitial retinal fluid undetectable to fluorescein angiography testing. Obtained results are in agreement with previously published data [9–11], which presented the visual acuity improvement and foveal structure normalization after dorzolamide treatment.

For the 1st time, mfERG was used to evaluate foveal function during the topical dorzolamide treatment of macular cysts in ESCS. MfERG is a well-known technique reflecting functional mapping of the retina including foveal region, especially bipolars and cone photoreceptors [21]. To date, to estimate the macular cysts treatment efficacy of ESCS patients visual acuity, retinal thickness measurements (OCT), contrast sensitivity and microperimetry were used [9–11]. However, visual acuity examination is not an accurate function test. The results of visual acuity measurements are subjective and allow only to estimate retinal function of about 1 central angle degree. Hence, visual acuity examination is not precise enough to estimate retinal function from retinal regions covering macular cysts, which usually surpasses 1 angle degree. That is why we decided to evaluate the retinal function with mfERG. Results of mfERGs provided in this case useful information that visual acuity and foveal thickness improvement corresponded with partial increase in foveal function. Assessment of foveal region function with mfERG was previously described in patients with epiretinal membrane, retinitis pigmentosa, diabetic macular edema, uveitic macular edema and others [22–24].

The results of our study suggest that the use of topical form of carbonic anhydrase inhibitor should be consider for a treatment of macular cysts associated with ESCS.
